# Supplementary material for: Mutations in PCYT2 disrupt etherlipid biosynthesis and cause a complex hereditary spastic paraplegia
Source: Brain. 2019 Oct 22;142(11):3382–97. doi: 10.1093/brain/awz291 (PMC6821184; doi:10.1093/brain/awz291)
Supplement: awz291_Supplementary_Data [file awz291_supplementary_data.zip › awz291-Suppl_data/Supplementary_Data4.zip!/Supplementary information 3 - fibroblast lipidomics.zip!/Fibroblast lipidomics/Controle vs Patient2/Statistical plots and data/PE species/Boxplots all.pdf]

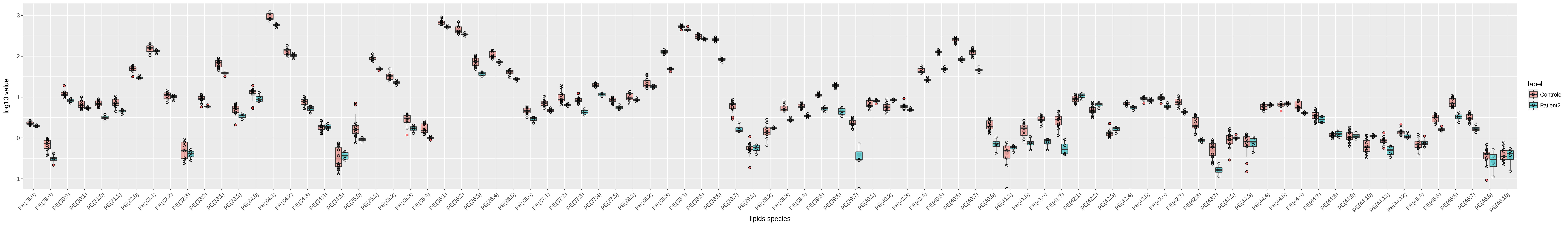
log10 value
3
2
1
0
-1
lipids species
PE(26:0) PE(29:0) PE(30:0) PE(30:1) PE(31:0) PE(31:1) PE(32:0) PE(32:1) PE(32:2) PE(32:3) PE(33:0) PE(33:1) PE(33:2) PE(34:0) PE(34:1) PE(34:2) PE(34:3) PE(34:4) PE(34:5) PE(35:0) PE(35:1) PE(35:2) PE(35:3) PE(35:4) PE(36:1) PE(36:2) PE(36:3) PE(36:4) PE(36:5) PE(36:6) PE(37:1) PE(37:2) PE(37:3) PE(37:4) PE(37:5) PE(38:1) PE(38:2) PE(38:3) PE(38:4) PE(38:5) PE(38:6) PE(38:7) PE(39:1) PE(39:2) PE(39:3) PE(39:4) PE(39:5) PE(39:6) PE(39:7) PE(40:1) PE(40:2) PE(40:3) PE(40:4) PE(40:5) PE(40:6) PE(40:7) PE(40:8) PE(41:1) PE(41:5) PE(41:6) PE(41:7) PE(42:1) PE(42:2) PE(42:3) PE(42:4) PE(42:5) PE(42:6) PE(42:7) PE(42:8) PE(43:7) PE(44:2) PE(44:3) PE(44:4) PE(44:5) PE(44:6) PE(44:7) PE(44:8) PE(44:9) PE(44:10) PE(44:11) PE(44:12) PE(46:4) PE(46:5) PE(46:6) PE(46:7) PE(46:8) PE(46:10)
label
Controle
Patient2
